# Conditional abrogation of *Atm* in osteoclasts extends osteoclast lifespan and results in reduced bone mass

**DOI:** 10.1038/srep34426

**Published:** 2016-09-28

**Authors:** Toru Hirozane, Takahide Tohmonda, Masaki Yoda, Masayuki Shimoda, Yae Kanai, Morio Matsumoto, Hideo Morioka, Masaya Nakamura, Keisuke Horiuchi

**Affiliations:** 1 Department of Orthopedic Surgery, Keio University School of Medicine, Tokyo 160-8582, Japan; 2 Japan Society for the Promotion of Science, Tokyo 102-8472, Japan; 3 Department of Anti-Aging Orthopedic Research, Keio University School of Medicine, Tokyo, Japan; 4 Department of Pathology, Keio University School of Medicine, Tokyo 160-8582, Japan

## Abstract

Ataxia-telangiectasia mutated (ATM) kinase is a central component involved in the signal transduction of the DNA damage response (DDR) and thus plays a critical role in the maintenance of genomic integrity. Although the primary functions of ATM are associated with the DDR, emerging data suggest that ATM has many additional roles that are not directly related to the DDR, including the regulation of oxidative stress signaling, insulin sensitivity, mitochondrial homeostasis, and lymphocyte development. Patients and mice lacking ATM exhibit growth retardation and lower bone mass; however, the mechanisms underlying the skeletal defects are not fully understood. In the present study, we generated mutant mice in which ATM is specifically inactivated in osteoclasts. The mutant mice did not exhibit apparent developmental defects but showed reduced bone mass due to increased osteoclastic bone resorption. Osteoclasts lacking ATM were more resistant to apoptosis and showed a prolonged lifespan compared to the controls. Notably, the inactivation of ATM in osteoclasts resulted in enhanced NF-κB signaling and an increase in the expression of NF-κB-targeted genes. The present study reveals a novel function for ATM in regulating bone metabolism by suppressing the lifespan of osteoclasts and osteoclast-mediated bone resorption.

Ataxia-telangiectasia mutated (ATM) is a 350 kDa Ser/Thr protein kinase that exists basally as an inactive dimer. Upon DNA damage, ATM is recruited to the damaged site and activated to initiate a cellular signal that regulates cell cycle checkpoints and promotes DNA repair^1^,^2^,^3^. The activation of ATM also leads to cellular senescence and apoptosis through the phosphorylation of p53 under certain conditions. Consistent with this, patients with mutations in the *ATM* gene, which cause a rare inherited disorder called ataxia-telangiectasia (A-T), are highly susceptible to DNA damage caused by ionizing radiation and exhibit various defects, including cerebellar ataxia, premature aging, and increased predisposition to malignancy^4^,^5^,^6^. While ATM is best characterized as a master regulator of the DNA damage response (DDR), emerging data have demonstrated that ATM has extensive functions in maintaining cellular homeostasis that are not directly related to the DDR. These additional roles include the regulation of oxidative stress^7^, metabolic signaling pathways (including insulin sensitivity and glucose metabolism)^8^,^9^,^10^,^11^, mitochondrial homeostasis^12^,^13^, immunity (most importantly, V(D)J recombination and class switch recombination)^14^,^15^,^16^,^17^,^18^,^19^, gonadal and germline development^14^,^20^,^21^,^22^, and telomere elongation^23^, among others. These observations clearly underscore the crucial functions of ATM not only in managing the DDR but also in regulating development and homeostasis in various types of cells and tissues.

Although A-T patients develop growth retardation, there are only a few studies describing the potential function of ATM in skeletal development. In the growth plates, ATM indirectly regulates chondrocyte proliferation and differentiation by regulating the amount of reactive oxygen species (ROS)^24^. The systemic abrogation of *Atm* in mice also results in decreased bone mass, most likely due to a steroid hormone deficiency caused by gonad developmental defects^20^,^25^. Whereas these studies show that mice lacking ATM exhibit reduced bone formation and increased bone resorption, the potential cell-autonomous functions of ATM in osteoblasts and osteoclasts are not yet fully understood.

Osteoclasts are multinucleated cells that are essential for bone resorption and remodeling *in vivo*^26^,^27^,^28^. The progenitors of osteoclasts are derived from the monocyte/macrophage cell lineage and express the receptor activator of NF-κB (RANK). Binding of RANK with RANK ligand (RANKL), a membrane-bound ligand expressed on osteoblasts and osteocytes, triggers intracellular signaling, which ultimately leads to osteoclast differentiation. Past studies have revealed that the differentiation and activity of osteoclasts are tightly regulated by various hormones and cytokines produced by immune cells and osteoblasts. However, despite the intricate mechanisms involved in maintaining skeletal homeostasis *in vivo*, the activity of osteoclasts is often dysregulated, resulting in overt bone resorption and reduced bone mass, as observed in skeletal disorders such as osteoporosis, bone metastasis, and arthritic bone destruction^29^,^30^,^31^. Therefore, it is clinically relevant and crucial to learn more about the regulatory mechanisms behind the differentiation and activity of osteoclasts.

In the present study, we generated mutant mice in which *Atm* is specifically abrogated under the control of the cathepsin K promoter (*Atm*^*Ctsk*^ mice) to elucidate the potential function of ATM in regulating osteoclast activity. These mice did not exhibit overt abnormalities but showed reduced bone mass due to increased bone resorption. Osteoclasts lacking ATM were more resistant to apoptosis and had an extended lifespan compared to control cells. Of note, the NF-κB pathway, a critical regulator of osteoclast activity and longevity^32^, was significantly enhanced in osteoclasts derived from *Atm*^*Ctsk*^ mice compared to those from control mice. These results show an unexpected role of ATM as a negative regulator of osteoclast longevity through suppressing NF-κB signaling *in vivo* and may shed novel light on the biology of ATM and skeletal homeostasis.

## Results

### *Atm* ^*Ctsk*^ mice exhibit reduced bone volume

**Figure 1:**
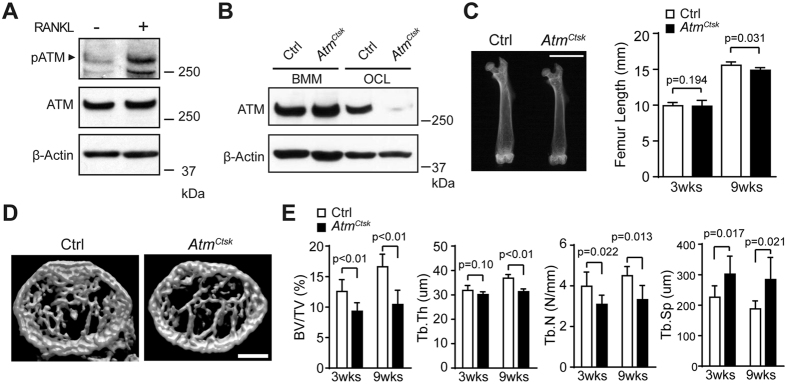
Conditional inactivation of ATM in osteoclasts results in decreased bone mass. (**A**) Western blots showing Phosphorylated-ATM (p-ATM) and total ATM in BMMs incubated with (+) or without (−) sRANKL for 5 d. Representative images of 3 independent experiments are shown. (**B**) Immunoblots of the cell lysates collected from BMMs and osteoclasts (OCL) probed for ATM. (**C**) X-ray images of the femurs of 9-week-old Ctrl and *Atm*^*Ctsk*^ mice (left panel). Scale bar, 5 mm. The lengths of the femurs collected from 3- and 9-week-old Ctrl and *Atm*^*Ctsk*^ mice are shown (right panel). (**D**) Reconstructed three-dimensional μCT images of the distal femurs of Ctrl and *Atm*^*Ctsk*^ mice. Scale bar, 500 μm. (**E**) Bone morphometric analysis of the femurs collected from 3- and 9-week-old Ctrl and *Atm*^*Ctsk*^ mice. BV/TV, bone volume/tissue volume; Tb.Th, trabecular thickness; Tb.N, trabecular number; Tb.Sp, trabecular space. n = 7 and 6 for 3-week-old Ctrl and *Atm*^*Ctsk*^mice, respectively, and n = 5 mice for each genotype at 9 weeks old.

In a preliminary experiment, we found that ATM is activated at a higher level in mature osteoclasts than in bone marrow-derived macrophages (BMMs), indicating that ATM is involved in a later stage of osteoclastogenesis or in the maintenance of osteoclast functions (Fig. 1A). The activation of ATM in mature osteoclasts was, in part, dependent on ROS production as a treatment with a potent ROS scavenger N-acetyl cysteine suppressed the phosphorylation of ATM (Supplementary Fig. 1A). On the other hand, DDR was not markedly induced in osteoclasts under the present experimental settings (Supplementary Fig. 1B). These results suggest that the activation of ATM is, at least in part, dependent on ROS production but not derived from DSB in mature osteoclasts.

**Figure 2:**
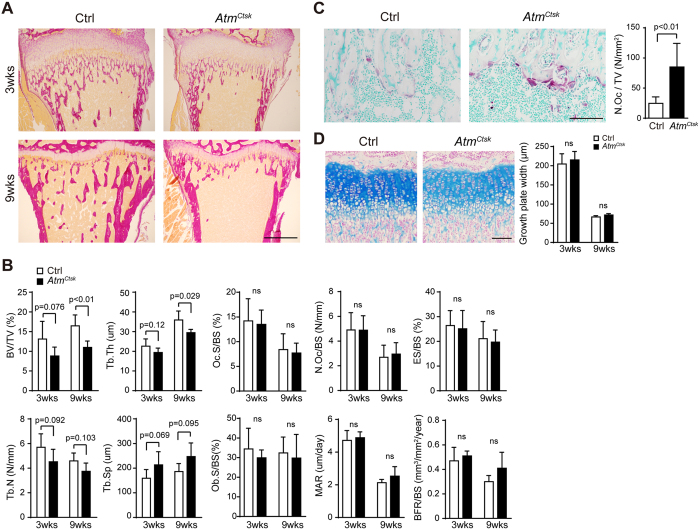
*Atm*^*Ctsk*^ mice have increased osteoclast numbers. (**A**) Sections of the proximal tibia of 3-week- (upper panel) and 9-week-old (lower panel) Ctrl and *Atm*^*Ctsk*^ mice stained with van Gieson stain. Scale bar: 500 μm. (**B**) Bone histomorphometry of the proximal tibia. BV/TV, bone volume/tissue volume; Tb.Th, trabecular thickness; Tb.N, trabecular number; Tb.Sp, trabecular space; Oc.S/BS, osteoclast surface/bone surface; N.Oc/BS, number of osteoclasts/bone surface; ES/BS, erosion surface/bone surface; Ob.S/BS, osteoblast surface/bone surface; MAR, mineral apposition rate; BFR/BS, bone formation rate/bone surface. (**C**) Sections of the tibia (3-week-old mice) stained for TRAcP and counterstained with methyl green. Images of the primary spongiosa are shown. Scale bar, 100 μm. The number of osteoclasts/tissue volume (N.Oc/TV) at the edge of tibial primary spongiosa of 3-week-old mice. (**D**) Sections of the tibia (3-week-old mice) stained with Alcian blue. Images of the growth plate are shown. Scale bar, 100 μm. Growth plate width of 3-week- (upper panel) and 9-week-old Ctrl and *Atm*^*Ctsk*^ mice was measured. n = 7 and 6 for 3-week-old Ctrl and *Atm*^*Ctsk*^ mice, respectively, and n = 5 mice for each genotype at 9 weeks old. ns, not significant.

To elucidate the potential role of ATM in osteoclasts, we generated mutant mice in which *Atm* is abrogated under the control of a *Ctsk* (encodes the osteoclast-specific proteolytic enzyme cathepsin K) promoter. *Atm*^*Ctsk*^ mice did not show any gross defects under unchallenged conditions (data not shown). *Atm*^*flox*/+^, *Atm*^*flox*/*flox*^, and *Atm*^*flox*/+^/*Ctsk-Cre* mice exhibited no apparent pathological phenotype and were used as control (Ctrl) animals in the present study. Western blot analysis showed that ATM was expressed in both BMMs and mature osteoclasts from Ctrl mice. The expression of ATM was also observed in *Atm*^*Ctsk*^ mouse-derived BMMs; however, this expression was markedly suppressed when the cells were induced into osteoclasts, demonstrating that the *Atm* alleles were successfully excised specifically in osteoclasts (Fig. 1B). Although *Atm*^*Ctsk*^ mice showed no significant difference in body size or weight (data not shown), the length of their long bones was approximately 5% shorter compared to Ctrl mice at 9 weeks of age (Fig. 1C). We next performed micro-computed tomography (μCT) analysis and found that *Atm*^*Ctsk*^ mice exhibited reduced bone mass compared to Ctrl mice, as highlighted by decreased BV/TV, Tb.Th, and Tb.N, and increased Tb.Sp values (Fig. 1D,E). Structural histomorphometric parameters (BV/TV, Tb.Th, Tb.N, and Tb.Sp) of the tibial sections at the secondary spongiosa showed a similar trend (Fig. 2A,B). Notably, we observed a significant increase in the osteoclast number in *Atm*^*Ctsk*^ mice at the edge of primary spongiosa (Fig. 2D). On the other hand, there was no significant differences in the bone formative or resorptive parameters in histomorphometric, inducing Oc.S/BS, Oc.N/BS, ES/BS, Ob.S/BS, MAR, and BFR/BS, in the secondary spongiosa (Fig. 2B), or in the thickness of the growth plates (Fig. 2D). These observations indicate that *Atm*^*Ctsk*^ mice exhibit reduced bone mass primarily due to an increase in osteoclastic bone resorption at the edge of primary spongiosa, whereas bone resorption at the secondary spongiosa is not overtly enhanced.

### BMMs derived from *Atm* ^*Ctsk*^ mice exhibit no apparent defects in differentiation

**Figure 3:**
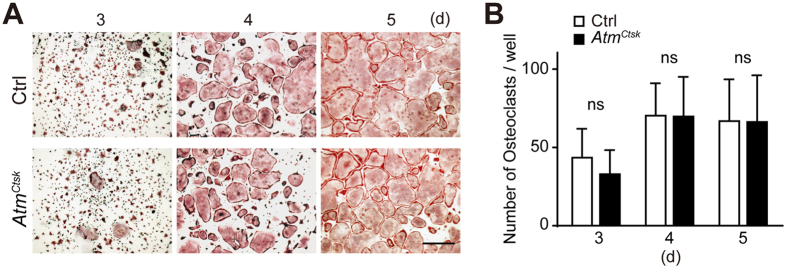
BMMs prepared from *Atm*^*Ctsk*^ bone marrow cells show no defect in osteoclast differentiation. (**A**) BMMs prepared from Ctrl and *Atm*^*Ctsk*^ bone marrow cells were incubated for 3, 4, or 5 d and stained for TRAcP. Scale bar: 1 mm. (**B**) The number of TRAcP-positive multinucleated cells in each well. n = 5 and 6 replicates for Ctrl and *Atm*^*Ctsk*^, respectively. ns, not significant.

The lower bone mass and increased osteoclast number observed in *Atm*^*Ctsk*^ mice at the primary spongiosa suggested that osteoclastogenesis was enhanced in these mice. BMMs collected from Ctrl and *Atm*^*Ctsk*^ mice were incubated in the presence of soluble RANKL (sRANKL) and macrophage colony-stimulating factor (M-CSF) for 3–5 d and stained for tartrate-resistant acid phosphatase (TRAcP). Contrary to our expectations, there was no difference in the number of multinucleated osteoclasts between Ctrl and *Atm*^*Ctsk*^ mouse-derived BMMs at any time period (Fig. 3). We also found no changes in the expression levels of genes involved in osteoclastogenesis (including *Nfatc1*, *Fos*, *Acp5*, and *Dcstamp*) between Ctrl and *Atm*^*Ctsk*^ osteoclasts (data not shown). These observations show that conditional abrogation of *Atm* under the control of the *Ctsk* gene promoter does not have any marked impact on osteoclast differentiation *in vitro*, as has been previously described^20^,^25^.

### Osteoclasts lacking *ATM* exhibit prolonged survival

**Figure 4:**
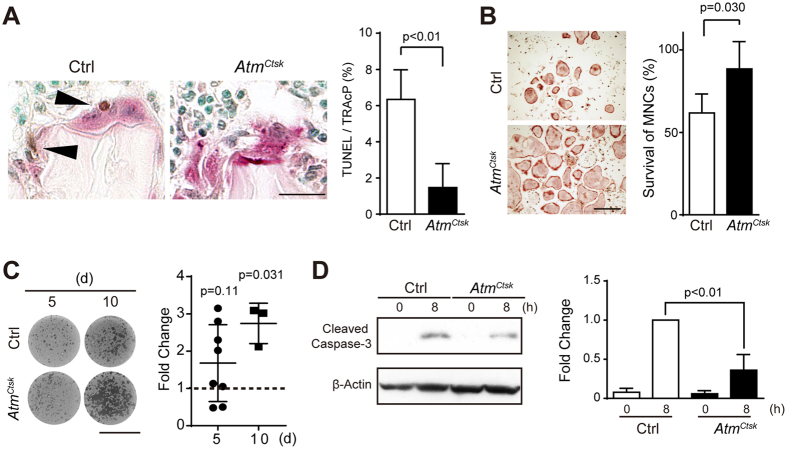
Osteoclasts lacking ATM are less prone to apoptosis. (**A**) Tibial sections from 3-week-old Ctrl and *Atm*^*Ctsk*^ mice dually stained for TUNEL and TRAcP (left panel). The ratio of TUNEL-positive osteoclasts in the tibial sections of 3-week-old control and *Atm*^*Ctsk*^ mice is shown (right panel). Scale bar: 20 μm. n = 7 for control and 6 for *Atm*^*Ctsk*^ mice, respectively. At least 50 osteoclasts were evaluated in each genotype. (**B**) Representative images of TRAcP-stained osteoclasts (left panel) and the ratio of remaining osteoclasts (right panel) 8 h after the withdrawal of sRANKL and M-CSF. The number of osteoclasts before the withdrawal of growth factors was set to 100%. n = 4 and 5 replicates for Ctrl and *Atm*^*Ctsk*^, respectively. Scale bar: 0.5 mm. (**C**) Representative images of osteoclast-formed pits (left) and the ratio of the pit area between Ctrl and *Atm*^*Ctsk*^ cells (right) on days 5 and 10 after sRANKL stimulation are shown. n = 8 and 3 for day 5 and 10, respectively. Each value was calculated from of 3-4 replicates. (**D**) Immunoblots for cleaved caspase-3 in Ctrl and *Atm*^*Ctsk*^ osteoclasts 0 and 8 h after the withdrawal of growth factors (left panel). Quantification of the relative changes in the signal intensity of cleaved caspase-3 (right panel). The signal intensity of cleaved caspase-3 in Ctrl cells 8 h after the starvation was normalized to 1. The data were obtained from 3 independent experiments.

Because BMMs derived from *Atm*^*Ctsk*^ mice were not defective in osteoclast differentiation, we next asked if the lack of ATM had any impact on the lifespan of osteoclasts. Tibial sections prepared from Ctrl and *Atm*^*Ctsk*^ mice were stained for apoptotic cells using the terminal deoxynucleotidyl transferase dUTP nick-end labeling (TUNEL) method and also stained for TRAcP. We enumerated the TRAcP-positive cells and assessed whether these cells contained nuclei that were positive for TUNEL. As shown in Fig. 4A, we found that the ratio of TRAcP-positive multinucleated cells containing at least one TUNEL-positive nucleus was significantly higher in *Atm*^*Ctsk*^ mice than in Ctrl mice, indicating that osteoclasts in *Atm*^*Ctsk*^ mice are less prone to apoptosis than those in Ctrl mice. To confirm this finding, we next induced osteoclasts *in vitro* using BMMs prepared from Ctrl and *Atm*^*Ctsk*^ mice and further cultured these cells under sRANKL- and M-CSF-depleted conditions for 8 h to induce apoptosis. At the end of the incubation, the cells were stained for TRAcP, and the number of surviving cells was enumerated. As shown in Fig. 4B, we found that more TRAcP-positive multinucleated cells remained on the plates with *Atm*^*Ctsk*^ mouse-derived cells than with control cells, indicating that osteoclasts derived from *Atm*^*Ctsk*^ BMMs were more resistant to growth factor-deprived conditions. In parallel with these findings, the amount of resorption, as evaluated with a pit formation assay, was increased in *Atm*^*Ctsk*^ osteoclasts compared to Ctrl osteoclasts, which was statistically significant at day 10 of incubation (Fig. 4C). This observation indicates that a lack of ATM in osteoclasts does not enhance the bone resorption activity *per se*, but the prolonged lifespan results in an overall increase in the amount of resorption. Furthermore, the protein level of cleaved caspase-3, a marker for apoptotic cells, was decreased in *Atm*^*Ctsk*^ cells compared to control cells under growth factor-deprived conditions, suggesting that osteoclasts lacking ATM are more resistant to apoptosis under the current experimental conditions (Fig. 4D).

### NF-κB activity is enhanced in osteoclasts lacking ATM

**Figure 5:**
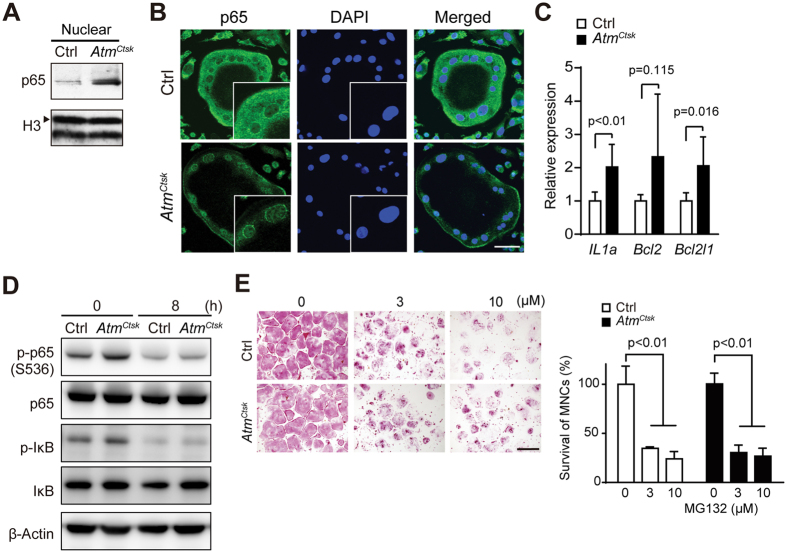
Enhanced NF-κB signaling in osteoclasts lacking ATM. (**A**) Immunoblots of the nuclear extracts of Ctrl and *Atm*^*Ctsk*^ osteoclasts probed for p65. Representative images of 3 independent experiments are shown. The blots for histone H3 (H3, arrowhead) serve as an internal control. (**B**) Ctrl and *Atm*^*Ctsk*^ osteoclasts stained for p65 and DAPI. The insets show a magnified image of the nuclei. Scale bar, 50 μm. (**C**) Relative expression of the transcripts of NF-κB target genes determined by quantitative PCR. n = 6 replicates. The average expression levels of each transcript in the Ctrl sample are set to 1. (**D**) Immunoblots for p65, IκB and their phosphorylated forms in Ctrl and *Atm*^*Ctsk*^ osteoclasts 0 and 8 h after the withdrawal of growth factors. Representative images of 3 independent experiments are shown. (**E**) TRAcP staining of Ctrl and *Atm*^*Ctsk*^ osteoclasts treated with MG-132 (0, 3, and 10 μM). The number of TRAcP-positive multinucleated cells in each well. n = 4 replicates for Ctrl and *Atm*^*Ctsk*^, respectively. Scale bar, 500 μm.

To further elucidate the potential mechanism behind the pro-apoptotic effect of ATM in osteoclasts, we next asked whether the lack of ATM in osteoclasts affects the activity of the NF-κB pathway, a signaling pathway critically involved in osteoclast survival^28^,^32^. Western blots using nuclear extracts from osteoclasts showed an increase in the level of p65 (encoded by *Rela*), one of the major components of the NF-κB complex, in *Atm*^*Ctsk*^ osteoclasts compared to Ctrl osteoclasts (Fig. 5A). Consistent with this, immunostaining for p65 revealed nuclear accumulation of p65 in *Atm*^*Ctsk*^ osteoclasts, which reflects increased NF-κB activity in the absence of ATM (Fig. 5B). Furthermore, there was an increase in the transcripts for anti-apoptotic genes, including *Il1a*, *Bcl2*, and *Bcl2l1* (encodes Bcl-XL), which are all regulated by the NF-κB pathway (Fig. 5C). In parallel with these findings, we also confirmed the increased levels of phosphorylated-IκB and phosphorylated-p65 (Ser536) (Fig. 5D, both of which reflect the activity of the NF-κB pathway. Of note, we found that the levels of both phosphorylated-IκB and phosphorylated-p65 (Ser536) decreased after sRANKL- and M-CSF-depletion, yet the expression of these molecules remained higher in *Atm*^*Ctsk*^ osteoclasts than in Ctrl cells (Fig. 5D). In addition, as have been previously described^33^,^34^, inhibition of p65 activity by a proteasome inhibitor MG-132 significantly suppressed the survival of both *Atm*^*Ctsk*^ and Ctrl osteoclasts (Fig. 5E), indicating that the survival of osteoclasts are highly dependent on the activity of the NF-κB pathway. Taken together, these observations suggest that the abrogation of *Atm* in osteoclasts extends the lifespan of osteoclasts most likely by enhancing NF-κB activity.

## Discussion

Our data show that the conditional abrogation of *Atm* in osteoclasts leads to decreased bone mass, potentially due to prolonged osteoclast survival. We found that fewer osteoclasts in *Atm*^*Ctsk*^ mice stained positive for TUNEL compared to those in Ctrl mice, indicating that a lack of ATM renders osteoclasts less prone to apoptosis. Consistently, osteoclasts from *Atm*^*Ctsk*^ mice were more resistant to apoptosis under sRANKL- and M-CSF-deprived conditions than those from Ctrl mice. Furthermore, we found that nuclear accumulation of p65 was enhanced in *Atm*^*Ctsk*^ osteoclasts, suggesting that the prolonged longevity of *Atm*^*Ctsk*^ osteoclasts was due to increased activation of the NF-κB pathway. Taken together, the present study reveals an unexpected role for ATM in regulating osteoclast longevity and bone volume.

The observation that the loss of ATM promotes the survival of mature osteoclasts was somewhat unexpected. Because ATM plays a protective role against genomic damage and the accumulation of intracellular ROS, we initially assumed that osteoclasts lacking ATM would be more prone to apoptosis and hence would have a shorter lifespan. In the developing central nervous system, radiation-induced cell death is largely dependent on ATM activity, and it has been shown that the loss of ATM renders neurons more resistant to apoptosis^35^. In a similar vein, our data suggest that the induction of apoptosis under physiological conditions is in part dependent on ATM activity in osteoclasts. However, a lack of ATM in osteoclasts does not have marked effects on the efficiency of bone resorption under physiological conditions in adult mice.

As a potential mechanism behind the enhanced survival of osteoclasts lacking ATM, we observed an increase in the activity of NF-κB in *Atm*^*Ctsk*^ osteoclasts. Because the activation of NF-κB can be triggered by ATM^36^,^37^, our data may appear counterintuitive. Nonetheless, a recent study has shown that the loss of ATM can induce NF-κB signaling, at least in certain cell types. Hathcock *et al*. demonstrated that systemic abrogation of ATM promotes the development of B-cell lymphoma in mice lacking T cells and that the NF-κB-pathway was significantly enhanced in these lymphoma cells^38^. The study also showed that the survival of the ATM-deficient lymphoma cells was dependent on NF-κB activity using pharmacological inhibitors of IKK. Although it is not fully understood how the loss of ATM enhances NF-κB activity in lymphocytes, the study supports the idea that ATM can both positively and negatively regulate NF-κB activity in a context- or cell-dependent manner. Given that the primary function of ATM is to induce DDR upon DNA double-strand break, it is also possible that the extended life-span was derived from the failure to induce DDR-mediated apoptosis. However, our data indicate that DDR is not noticeably induced in mature osteoclasts (Supplementary Fig. 1B), arguing against this hypothesis.

How ATM is activated in mature osteoclasts and how ATM negatively regulates the activity of NF-κB remain to be addressed. Our data suggest that ROS production is partially responsible for the activation of ATM^39^. On the other hand, we did not find any marked DDR in mature osteoclasts, indicating that DSB is not the major contributor for ATM activation. The potential mechanisms behind the suppression of NF-κB activity by ATM may include the inhibition of p65 activity via the phosphorylation of Ser547 by ATM^40^; the reciprocal inhibition of NF-κB and p53, where the abrogation of the ATM-p53 axis reversely enhances NF-κB activity^41^; the competitive binding of ATM and NF-κB to protein phosphatase 2A, which exhibits inhibitory activity towards both ATM and NF-κB^42^,^43^. In the present study we found the levels of the phosphorylation of p65 (Ser536) and IκB were increased in *Atm*^*Ctsk*^ osteoclasts compared to that in Ctrl (Fig. 5D). This observation indicates that the NF-κB pathway is activated through the conventional pathway which involves phosphorylation and degradation of IκB in *Atm*^*Ctsk*^ osteoclasts. On the other hand, it remains to be clarified what the direct substrates of ATM in osteoclasts and whether other factors than phosphorylation of IκB and p65 are involved in the activation of the NF-κB pathway in osteoclasts. Screening of the potential ATM substrates in osteoclasts using mass spectrometry may help to address these issues.

There are limitations in the present study. First, most importantly, because all of the animal experiments were performed under unchallenged conditions, it is not clear whether the results of the present study reflect the pathological conditions of skeletal diseases with enhanced bone resorption. Second, because we used mice of mixed genetic background (129Sv and C57BL/6) for morphometric analyses the data potentially have a large dispersion. Nevertheless, the data from 3 week-old mice showed a similar trend with those of 9 week-old mice, indicating that our results were consistent among different sets of animals. Of note, a recent study has shown that the *Atm* protein and transcript levels decline with aging in splenocytes^44^. Accordingly, our preliminary observations indicate that this is also the case for bone marrow cells and osteoclasts (data not shown). Because a loss of ATM in osteoclasts results in enhanced bone resorption, it is tempting to speculate that the decrease in ATM activity is causally related to the pathogenesis of senile osteoporosis.

In summary, the present study identifies ATM as a novel negative regulator of osteoclastic bone resorption. The loss of ATM in osteoclasts leads to enhanced NF-κB activity and promotes their survival, leading to an increase in overall bone resorption. Our data therefore reveal an unexpected role for ATM, a critical molecule involved in the maintenance of genome integrity, in osteoclast longevity and bone resorption.

## Methods

### Mice

The *Atm*^*flox*/*flox*^ mice and *Ctsk-Cre* transgenic mice were generated as previously described^16^,^45^. The mice used in the present study are of a mixed genetic background (129Sv and C57BL/6). All animal experiments were approved by the Institutional Animal Care and Use Committee of the Keio University School of Medicine (approval number, 09101) and performed in accordance with the approved guidelines. *Atm*^*flox*/*flox*^ mice and *Ctsk-Cre* transgenic mice were kindly provided by Dr. Fred Alt (Harvard Univ.) and Dr. Shigeaki Kato (Tokyo Univ.), respectively. Histology and morphometric analyses were performed using male mice. Bone marrow cells were collected from both male and female mice.

### Reagents and antibodies

Anti-ATM (2C1), anti-p65 (C-20), anti-p53 (FL-393), and anti-β actin (C4) antibodies were obtained from Santa Cruz Biotechnology (Dallas, TX). Anti-cleaved caspase-3 (Asp175, 9661), anti-histone H3, anti-phosphorylated p65 (Ser536, 3031), anti- phosphorylated IκB (Ser32/36, 9246), anti-phosphorylated p53 (Ser15, 9284), anti-phosphorylated H2AX (Ser139, 9718), and anti-IκB antibodies were purchased from Cell Signaling Technology (Danvers, MA). Anti-phospho-ATM (Ser1981) antibody (10H11.E12) was obtained from Rockland Immunochemicals (Limerick, PA). To detect phospho-ATM, blocking was performed using phosphoBLOCKER Blocking Reagent (Cell Biolabs, San Diego, CA). M-CSF and sRANKL were purchased from Kyowa Kirin (Tokyo, Japan) and PeproTech (Rocky Hill, NJ), respectively.

### μCT and histomorphometric analysis

Three-dimensional images of the distal femurs were reconstructed using a μCT scanner (R_mCT2, Rigaku, Yokohama, Japan) and the 3D software TRI/3D-BON (Ratoc System Engineering, Tokyo, Japan). The ethanol-fixed tibiae were embedded in glycol methacrylate resin and sectioned into 5-μm slices. For histomorphometric analyses, an area 1.2 mm above the growth plate (1.62–2.34 mm^2^ in size) at the proximal metaphysis was evaluated. For the assessment of osteoclasts, tibial sections were stained for TRAcP and with van Gieson’s solution and Alcian blue. All of the measurements were performed in a blinded manner.

### Osteoclast culture

BMMs were prepared as previously described^46^ and used as osteoclast precursors. The BMMs were cultured in the presence of sRANKL (50 ng/ml) and M-CSF (3.3 × 10^3^ U/ml) for 5 d to induce osteoclastogenesis. TRAcP staining was performed as previously described^46^. For the osteoclast survival assay, osteoclasts induced *in vitro* were cultured for 8 h in the absence of sRANKL and M-CSF as previously described^47^,^48^. At the end of the incubation, the cells remaining on the dishes were stained for TRAcP, and the TRAcP-positive multinucleated cells (>3 nuclei) were enumerated. To examine the inhibitory effect of MG-132 on osteoclast survival, mature osteoclasts formed *in vitro* were treated with MG-132 (0, 3, and 10 μM) for 18 h. The surviving cells were fixed and stained for TRAcP.

### RNA isolation and quantitative RT-PCR

Total RNAs were extracted from cells using Sepasol reagent (Nacalai Tesque, Kyoto, Japan) and used for RT-PCR analyses. The nucleotide sequences of the oligos used in the present study are presented in Supplementary Table 1. The relative gene expression was determined by SYBR Green–based real-time PCR using a 7300/7500 Fast real-time PCR system (Applied Biosystems, Foster City, CA) and a LightCycler II (Roche, Risch-Rotkreuz, Switzerland). The expression levels were normalized to the *Gapdh* transcript expression levels.

### Resorption pit assay

Osteoclast activity was evaluated using an Osteo Assay Surface (Corning, Corning, NY) according to the manufacturer’s instructions. Stripwells were scanned using a flatbed scanner, and osteoclast-formed pits were analyzed using ImageJ software (http://imagej.nih.gov/ij/index.html).

### Immunofluorescence microscopy

The cells were fixed with 4% paraformaldehyde for 15 min and permeabilized in 0.3% Triton X-100 for 10 min. The fixed cells were incubated with a primary antibody overnight at 4 °C and subsequently with a secondary antibody (AlexaFluor 488–conjugated anti-rabbit IgG or AlexaFluor 546/555–conjugated anti-mouse IgG, Invitrogen, Carlsbad, CA) for 1 h at 37 °C. The nuclei were stained with DAPI (Vectashield HardSet Mounting Medium with DAPI, Vector Laboratories, Burlingame, CA). Images were captured using a FluoView FV10i confocal microscope (Olympus, Tokyo, Japan) and the Olympus FluoView Viewer software (Ver. 3.1). The acquired images were processed with Adobe Photoshop CS6 (Adobe Systems, San Jose, CA) for contrast and black balance correction.

### Evaluation of apoptotic multinucleated cells

The sections of the tibiae collected from 3-week-old *Atm*^*Ctsk*^ and Ctrl mice were stained for TRAcP and TUNEL. TUNEL staining was performed using an *In situ* Apoptosis Detection Kit (Takara Bio, Shiga, Japan) according to the manufacturer’s instructions. The nuclei were counterstained with methyl green. At least 50 TRACP-positive osteoclasts in each section were evaluated for the presence of TUNEL-positive nuclei, and the ratio of TUNEL-positive multinucleated cells was calculated.

### Statistics

All statistical analyses were performed using GraphPad Prism 7 (GraphPad Software, La Jolla, CA). Two-tailed Student’s *t*-tests for 2 samples assuming equal variances were used to calculate the p values except for those in Fig. 5E. One-way ANOVA was used to calculate the p values in Fig. 5E. Values of p < 0.05 were considered significant. The values are presented as the mean ± SD.

## Additional Information

**How to cite this article**: Hirozane, T. *et al*. Conditional abrogation of *Atm* in osteoclasts extends osteoclast lifespan and results in reduced bone mass. *Sci. Rep.* **6**, 34426; doi: 10.1038/srep34426 (2016).

## Supplementary Material

Supplementary Information
