# Supplementary material for: Conditional abrogation of Atm in osteoclasts extends osteoclast lifespan and results in reduced bone mass
Source: Sci Rep. 2016 Sep 28;6:34426. doi: 10.1038/srep34426 (PMC5039636; doi:10.1038/srep34426)
Supplement: Supplementary Information [file srep34426-s1.pdf]

Supplementary Information

Conditional abrogation of *Atm* in osteoclasts extends osteoclast lifespan and results in reduced bone mass

Toru Hirozane [1,2], Takahide Tohmonda [1,3], Masaki Yoda [1,3], Masayuki Shimoda [4], Yae Kanai [4], Morio Matsumoto [1], Hideo Morioka [1], Masaya Nakamura [1], and Keisuke Horiuchi [1,3]

[1]Department of Orthopedic Surgery, Keio University School of Medicine, Tokyo 160-8582, Japan, [2]Japan Society for the Promotion of Science, Tokyo 102-8472, Japan, [3]Department of Anti-Aging Orthopedic Research, Keio University School of Medicine, Tokyo, Japan, [4]Department of Pathology, Keio University School of Medicine, Tokyo 160-8582, Japan

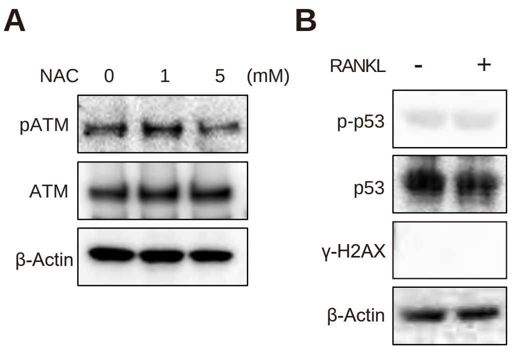

Supplementary Figure 1. ATM activation in mature osteoclasts is partly dependent on ROS production. (A) Immunoblots of phosphorylated-ATM (p-ATM) and total ATM in osteoclasts treated with N-acetyl cysteine (NAC; 0, 1, and 5 mM) for 6h. Immunoblots for β-actin were used as loading controls. (B) Western blots showing phosphorylated-p53 (p-p53), total p53, and γ-H2AX in BMMs incubated with (+) or without (-) sRANKL for 5 d.

Supplemental Table 1

List of oligonucleotides used for quantitative PCR analysis

Gene	Strand	Sequence
Atm	Fwd	CGTGATGACCTGAGGCAAGA
	Rev	GCTGAGAAAGGGGAACCACC
Il1a	Fwd	CGAAGACTACAGTTCTGCCATT
	Rev	GACGTTTCAGAGGTTCTCAGAG
Bcl2	Fwd	ATGCCTTTGTGGAACTATATGGC
	Rev	GGTATGCACCCAGAGTGATGC
Bcl2l1	Fwd	GCTGGGACACTTTTGTGGAT
	Rev	TGTCTGGTCACTTCCGACTG
Nfatc1	Fwd	AACGCCCTGCTGACCACCGATAGCA
	Rev	CCCGGGTGCCTTCCGTCTCATA
Fos	Fwd	CCAAGCGGAGACAGATCAACTT
	Rev	TCCAGTTTTTCCTTCTCTTTCAGCAGAT
Acp5	Fwd	CCAATGCCAAAGAGATCGCC
	Rev	TCTGTGCAGAGACGTTGCCAAG
Dcstamp	Fwd	TCCTCCATGAACAAACAGTTCCAA
	Rev	AGACGTGGTTTAGGAATGCAGCTC
Gapdh	Fwd	TCAACAGCAACTCCCACTCTTCC
	Rev	ACCCTGTTGCTGTAGCCGTATTC
